# Catheter Related Bloodstream Infection (CR-BSI) in ICU Patients: Making the Decision to Remove or Not to Remove the Central Venous Catheter

**DOI:** 10.1371/journal.pone.0032687

**Published:** 2012-03-05

**Authors:** Rodrigo Octávio Deliberato, Alexandre R. Marra, Thiago Domingos Corrêa, Marinês Dalla Vale Martino, Luci Correa, Oscar Fernando Pavão dos Santos, Michael B. Edmond

**Affiliations:** 1 Critical Care Unit, Hospital Israelita Albert Einstein, Sao Paulo, Brazil; 2 Microbiology Laboratory Department, Hospital Israelita Albert Einstein, Sao Paulo, Brazil; 3 Infection Control Unit, Hospital Israelita Albert Einstein, Sao Paulo, Brazil; 4 Department of Internal Medicine, Virginia Commonwealth University School of Medicine, Richmond, Virginia, United States of America; University of Cincinnati, United States of America

## Abstract

**Background:**

Approximately 150 million central venous catheters (CVC) are used each year in the United States. Catheter-related bloodstream infections (CR-BSI) are one of the most important complications of the central venous catheters (CVCs). Our objective was to compare the in-hospital mortality when the catheter is removed or not removed in patients with CR-BSI.

**Methods:**

We reviewed all episodes of CR-BSI that occurred in our intensive care unit (ICU) from January 2000 to December 2008. The standard method was defined as a patient with a CVC and at least one positive blood culture obtained from a peripheral vein and a positive semi quantitative (>15 CFU) culture of a catheter segment from where the same organism was isolated. The conservative method was defined as a patient with a CVC and at least one positive blood culture obtained from a peripheral vein and one of the following: (1) differential time period of CVC culture versus peripheral culture positivity of more than 2 hours, or (2) simultaneous quantitative blood culture with 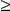
5∶1 ratio (CVC versus peripheral).

**Results:**

53 CR-BSI (37 diagnosed by the standard method and 16 by the conservative method) were diagnosed during the study period. There was a no statistically significant difference in the in-hospital mortality for the standard versus the conservative method (57% vs. 75%, p = 0.208) in ICU patients.

**Conclusion:**

In our study there was a no statistically significant difference between the standard and conservative methods in-hospital mortality.

## Introduction

Approximately 150 million central venous catheters (CVC) are used each year in the United States. These catheters have unquestionable benefits in current medical practice, but their potential complications are also well known [1].

One of the main complications is catheter-related bloodstream infection (CR-BSI). In the United States 150,000 new cases are estimated to occur each year, of which approximately 80,000 occur in intensive care units (ICUs). Each new episode of CR-BSI increases the risk of death by 12 to 25%, in addition to prolonging hospitalization and increasing healthcare costs by $30,000 to $50,000 [2], [3], [4].

In an attempt to reduce the rate of these infections, the Institute for Health Care Improvement (IHI) and the Centers for Disease Control and Prevention (CDC) have issued guidelines for the prevention of CR-BSI, including: hand hygiene, maximum barrier precautions for insertion, skin antisepsis with chlorhexidine, careful choice of the insertion site and a proactive approach to CVC removal [3], [5].

The correct diagnosis of the infection is as important as these recommendations. Clinical methods are known to have low sensitivity and specificity and the current standard method for diagnosis requires CVC removal and semi-quantitative culture of the CVC tip together with a peripheral blood culture [3]. Unfortunately, more than 70% of the suspected CR-BSIs yield negative blood culture results (no growth), meaning that the CVC was unnecessarily removed [4], [6], [7], [8].

Several conservative methods (not involving CVC removal) have been investigated over recent years with the objective of improving CR-BSI diagnostic accuracy for short-term catheters and as well to avoid patient exposure to the risks of a new CVC insertion [8].

Among the conservative methods described for diagnosing CR-BSI are the paired quantitative blood cultures from the CVC and peripheral vein and paired blood culture from the CVC and peripheral vein with differential time to positivity. Both methods have been shown to be reliable, both for the diagnosis of CR-BSI and for the identification of CVC colonization in patients with short-term catheters [6], [8].

So far, we are not aware of any study correlating suspected CR-BSI management methods with clinical outcomes. Therefore, this study was conducted with the objective of assessing the impact of the standard (CVC removal) versus the conservative (no CVC removal) methods on the clinical outcome (death) of ICU patients with CR-BSI.

## Methods

### Setting

This study was conducted in the ICU of a tertiary care, private hospital in São Paulo, Brazil. This open model ICU is a 38-bed medical-surgical unit where approximately 2,200 patients are admitted each year.

### Study Design

This was a retrospective study that reviewed all occurrences of bloodstream infection (BSI) in the ICU over the 9 year period January 1, 2000 to December 31, 2008.

These episodes were classified as catheter associated bloodstream infection (CABSI) and catheter related bloodstream infection (CR-BSI) [9]. Only the patients with CR-BSI were included in our study.

Patients over 18 years old with CR-BSI were included in the study and only the first episode of CR-BSI of each patient was included. Exclusion criteria was pregnancy. No written informed consent was required because it was a retrospective study. This study was approved by our Institutional Review Board (IRB) – The Ethical Committee of Hospital Israelita Albert Einstein.

The data collected included age, sex, admission date, date when bacteremia was identified, outcome date, as well as the SIRS, SAPS II, APACHE II, and SOFA scores [10], [11], [12], [13] from two days before the bacteremia until 14 days after the BSI event. Data related to the CVC, such as type, site, indwelling time, complications during insertion, the method used to diagnose CR-BSI (standard versus conservative), as well as data on the ICU admission diagnosis, prior antibiotic therapy, adjustments to antimicrobial therapy in the first 24 hours, Charlson score [14] and death (in-hospital) were also collected.

### Definitions

The use of the standard method (removal of the central venous catheter) for diagnosis of CR-BSI was defined as a patient with a CVC and at least one positive blood culture obtained from a peripheral vein and a positive semi-quantitative culture of a catheter segment (>15 CFU) from which the same organism (species and antimicrobial susceptibility) was isolated [7]. The use of the conservative method (without removal of the central venous catheter), to diagnose CR-BSI was defined as a patient with a CVC and at least one positive blood culture obtained from a peripheral vein plus one of the following: (1) differential time period of CVC blood culture versus peripheral blood culture positivity of more than 2 hours, (2) simultaneous quantitative blood culture with a ≥5∶1 ratio CFU (CVC to peripheral) [6]. Until December of 2003 the only way to make the CR-BSI diagnosis in our department was using the standard method. After that in 2004 the conservative methods started in our institution and unless the patient was seriously ill (hypotension, hypoperfusion or signs and symptoms of organ failure) this was the method of choice in patients with suspected CR-BSI [2]. As soon as we made the CR-BSI diagnosis (even using the conservative method), the central venous catheter was removed as part of the treatment.

In our institution the internal jugular vein has been the first choice for CVC placement since 2000. The catheters used were Arrows® 7 french - 20 cm, triple lumen and double lumen and Arrows® 12 french triple lumen - 20 cm (dialysis catheter). All procedures were done using the sterile techniques recommend by CDC (hand hygiene, skin antisepsis, aseptic technique, specific catheter site dressings regimens and since April 2007 a daily review of all lines with prompt removal of unnecessary lines) [3].

The clinical condition of each patient during the catheter-related bloodstream infection was assessed daily and rated as SIRS, sepsis, severe sepsis or septic shock using criteria previously published by the American College of Chest Physicians/Society of Critical Care Medicine (ACCP/SCCM) [10]. Systemic inflammatory response syndrome (SIRS) was defined by the presence of two or more of the following: (1) temperature ≥38.3°C or ≤36°C, (2) respiratory rate 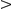
20 breaths per minute or a PaCO2 <32 mmHg, (3) heart rate >90 beats per minute or (4) white blood cell count >12×10^3^/µL or <4×10^3^/µL or the presence of more than 10% immature neutrophils [10].

Sepsis was defined as SIRS associated with at least one positive blood culture. Sepsis associated with organ dysfunction, hypotension or systemic manifestations of hypoperfusion constituted severe sepsis. Septic shock was defined as sepsis associated with hypotension unresponsive to intravenous fluid challenge or requiring a vasopressor agent [10].

The presence of organ system failure was assessed using the criteria described by Fagon [15]. Adequate empiric antimicrobial treatment was defined as antibiotic therapy administered within 24 hours after blood culture samples were obtained which the microorganism was susceptible [16].

The primary endpoint was overall in-hospital mortality and the secondary endpoints were ICU mortality, mortality in the conservative method group during the first 24 hours and after 24 hours while the CVC was kept in place.

### Microbiological methods

#### Standard method

The catheter tip culture was performed using the Maki method, in which a 5 cm segment of the catheter tip was rolled across a blood agar plate. The plate was then incubated at 37°C for 24 hours. Results were reported for growth equal to or exceeding 15 CFU/mL.

For the peripheral blood culture, 20 mL of blood were collected from a peripheral site and inoculated in BACTEC™ Plus Aerobic/F Medium and BACTEC™ Plus Anaerobic/F Medium bottles. The bottles were then incubated in the BD BACTEC™ 9240 System for up to 5 days.

#### Conservative method

Two methods were used: paired blood cultures with differential time to positivity and number of colonies count.

For this purpose up to 20 mL of blood were collected from a peripheral site and the same amount of blood was drawn from the central venous catheter.

The same amount of blood was inoculated on the BACTEC™Plus Aerobic/F Medium and HEMOBAC Trifásico™ (PROBAC do Brasil) media. The BACTEC™Plus Aerobic/F Medium bottle followed the same routine previously described; the HEMOBAC Trifásico™ system consists of a liquid phase coupled with a dip slide with chocolate agar, MacConkey agar, and Sabouraud agar, where colonies can be counted once there is bacterial growth.

### Statistical analysis

For continuous variables, mean values were compared using two-sample t-tests for independent samples. For continuous variables, median values were compared using the Mann-Whitney test. Differences in proportions were compared using a chi-squared test or Fisher s exact test when appropriate. Values are reported as mean ± SD. All significance tests are two-tailed. Variables significant for predicting mortality in univariate analysis were entered into a logistic regression model when p-value<0.1. When colinearity was identified between two variables, the one with the greatest clinical relevance associated with mortality was included in the multivariate analysis. The association of independent variables was expressed as odds with 95% confidence intervals. Alpha was set at 0.05. All statistical analyses were done using the Statistical Package for the Social Sciences software (SPSS, Chicago, IL, U.S.A.).

## Results

### Study population and patient characteristics

During the study period, a total of 247 bloodstream infections occurred in our ICU. Of those 192 were classified as catheter associated bloodstream infection and 55 were catheter-related bloodstream infections.

Two patients (of those 55 with CR-BSI) were excluded because they were transferred to other hospitals, so 53 were included in this study.

Patients included in this study had a mean age of 64±19.29 years. Fifty-three percent were male. At the onset of bacteremia, the APACHE II mean value ± standard deviation was 15.43±4.52. At ICU admission, the main diagnoses were severe sepsis (28%), respiratory failure (20%), post-operative (17%), other shock states (13%), coronary heart disease (10%), and neurologic disorders (10%) and acute renal failure (2%).

The main risk procedures before bacteremia were: mechanical ventilation (75%), use of vasoactive drugs (55%), parenteral nutrition (40%), hemodialysis (40%), and blood transfusions (17%). Concerning the CVC characteristics, 70% were double-lumen catheters, 26% hemodialysis catheter and 93% were inserted into the anterior internal jugular vein with an indwelling time (mean ± standard deviation) of 16.32±8.56 days. Of the 53 catheter-related bloodstream infections recorded in this study, 37 (69.8%) were diagnosed using the standard method (catheter removal). Eighty five percent of the patients had received antibiotic therapy over the 15-day period preceding bacteremia [**Table 1**]. For all cases in our conservative group the diagnosis was performed by differential time to positivity.

**Table 1:** Characteristics of the patients with CR-BSI.

	Standard methodN = 37	Conservative methodN = 16	p-value	OR 95% CI
**Demographic data**				
Age, mean ± SD	66.83±20.10	59.31±16.6	0.2	
Male gender	17 (45%)	11 (68%)	0.127	0.39 (0.11–1.33)
Parenteral nutrition	13 (35%)	08 (50%)	0.31	1.85 (0.56–6.07)
Indwelling bladder catheter	32 (86%)	14 (88%)	0.92	1.09 (0.19–6.33)
Vasoactive drug	22 (59%)	07 (44%)	0.292	0.53 (0.16–1.74)
Mechanical ventilation	31 (84%)	09 (56%)	0.032	0.25 (0.07–0.93)
Acute renal failure	23 (62%)	10 (62%)	0.981	1.01 (0.30–3.40)
Coagulation disorder	04 (11%)	04 (25%)	0.151	3 (0.64–14.06)
Dialysis	13 (35%)	08 (50%)	0.31	1.85 (0.56–6.07)
Prior antibiotic therapy	32 (86%)	13 (81%)	0.625	0.68 (0.14–3.25)
Adequate treatment in 1^st^ 24 hours	15 (40%)	07 (44%)	0.828	1.14 (0.35–3.73)
Lenght of hospital stay before bacteremia (days) median (range)	25 (3–245)	24.5 (9–143)	0.69	
Blood transfusion	12 (32%)	05 (31%)	0.933	0.95 (0.27–3.34)
Septic shock	26 (70%)	12 (75%)	0.726	1.27 (0.33–4.81)
**Measurement scores**				
APACHE II at admission, mean ± SD	12.16±5.29	11.88±7.81	0.89	
SAPS II at admission, mean ± SD	36.03±12.52	29.50±13.23	0.1	
SOFA at admission, mean ± SD	3.81±4.36	03.69±3.38	0.92	
APACHE II bacteremia, mean ± SD	15.43±4.62	15.44±4.43	0.99	
SAPS II bacteremia, mean ± SD	43.05±12.64	38.06±12.91	0.2	
SOFA bacteremia, mean ± SD	06.97±04.41	07.06±03.43	0.94	
Charlson >3	3.2±2.6	3.6±2.5	0.59	−0.41 (−1.96–1.14)
**CVC characteristics**				
CVC insertion site:				
Anterior jugular vein	33 (89.2%)	16 (100%)		
Subclavian vein	2 (5.4%)	-		
Femoral vein	1 (2.7%)	-		
External jugular vein	1 (2.7%)	-		
Type of CVC:				
Double lumen	29 (78.4%)	9 (56.3%)		
Dialysis catheter	1 (2.7%)	-		
Triple lumen	7 (18.2%)	7 (43.8%)		
CVC indwelling time (days), mean ± SD	17±9.2	14.8±6.8	0.35	2.16 (−2.43–6.75)
**Outcome**				
ICU mortality	17 (46%)	12 (75%)	0.051	3.52 (0.96–12.99)
In-hospital mortality	21 (57%)	12 (75%)	0.208	2.29 (0.62–8.43)

CVC = central venous catheter; SAPS II = simplified acute physiology score; SOFA = Sepsis-related Organ Failure Assessment; APACHE II = Acute Physiology and Chronic Health disease Classification System II, Charlson = comorbidity index.

### Microbiological features

Of the 53 cases of CR-BSI, 23 (43.4%) were due to gram-negative bacilli, 19 (35.9%) were due to fungi and 11 (20.7%) were caused by gram-positive cocci.

Of the gram-negative bacilli (43% *Acinetobacter baumannii*, 13% *Pseudomonas aeruginosa*, 13% *Klebsiella pneumoniae*, 13% *Enterobacter* spp and 18% other species); 35% were resistant to ciprofloxacin and ceftazidime and sensitive to imipenem, 30% were resistant to imipenem, ciprofloxacin and cefazidime, and 35% were susceptible to ciprofloxacin, ceftazidime and imipenem.

Of all fungal infections, 42% by *Candida albicans* (all susceptible to fluconazole), and the remainder were caused by non-albicans species.

Of the gram-positive cocci, 54% were coagulase-negative staphylococci, 10% were methicillin-susceptible *Staphylococcus aureus* (MSSA), and 36% were *Enterococcus faecalis* (all sensitive to vancomycin). There were no cases of methicillin-resistant *Staphylococcus aureus* (MRSA).

**Table 2** describes the microbiologic findings according to the diagnostic method.

**Table 2:** Microbiological features by diagnostic method.

	Conservative method16 cases	Standard method37 cases
**Gram negative bacilli**	**6 (100%)**	**17 (100%)**
*Acinetobacter baumannii*	2 (33.3%)	8 (47%)
*Burkhloderia cepacia*	1 (16.7%)	1 (5.9%)
*Pseudomonas aeruginosa*	1 (16.7%)	1 (5.9%)
*Proteus mirabilis*	-	1 (5.9%)
*Klebsiella pneumoniae*	-	2 (11.8%)
*Stenotrophomonas maltophila*	-	2 (11.8%)
*Enterobacter aerogenes*	-	2 (11.8%)
*E. coli*	1 (16.7%)	-
*Enterobacter clocae*	1 (16.7%)	-
**Gram positive cocci**	**3 (100%)**	**7 (100%)**
*Enterococcus faecalis*	1 (33.3%)	2 (28.6%)
*Staphylococcus aureus*	-	1 (14.3%)
*Staphylococcus epidermidis*	1 (33.3%)	4 (57.1%)
*Staphylococcus simulans*	1 (33.3%)	-
**Fungi**	**7 (100%)**	**13 (100%)**
*Candida albicans*	3 (42.9%)	6 (46.2%)
*Candida parapsilosis*	1 (14.3%)	5 (38.5%)
*Candida glabrata*	-	1 (7.7%)
*Candida krusei*	2 (28.6%)	-
*Candida tropicalis*	1 (14.3%)	-
*Saccharomyces cerevisiae*	-	1 (7.7%)

### Outcomes

The analysis showed that 33 (62.2%) of the 53 patients died during hospitalization, 21 (63.6%) in the standard method group and 12 (36.4%) in the conservative method group. Of the 29 deaths occurring in the ICU, 17 (58.6%) were from the conservative method group and 12 (41.3%) from the standard method group. All 53(100%) patients had received antimicrobial therapy based on the peripheral and CVC blood cultures, and 22 patients (41.5%) had their antibiotic regimen adjusted within the first 24 hours. There was no difference in in-hospital mortality between the groups (57% vs. 75% for standard vs. conservative, p = 0.208) or for other risk factors assessed (age, sex, parenteral nutrition, blood transfusion, indwelling bladder catheter, coagulation disorder, prior antibiotic therapy vasoactive drugs, mechanical ventilation, acute renal failure, dialysis, death in the ICU, SAPS II, SOFA and APACHE II at admission and at the onset of bacteremia, presence of septic shock, length of hospital stay before bacteremia, Charlson >3, cvc indwelling time) [**Table 1**].

Concerning secondary endpoints, the mortality rate in the first 24 hours was the same in the standard method group and in the conservative method group, 56%. However, when the standard method was compared to the conservative method after the CVC had been in place for 24 hours, different mortality rates were observed 56% versus 100%, respectively. Of the 16 cases of CR-BSI diagnosed using the conservative method, in 9 (56%) the catheter was removed within 24 hours maximum, in 4 (25%) it was removed within 48 hours and in 3 (18.9%) it was removed after 72 hours.

According to the univariate analysis, the specific variables such as age, renal dysfunction, hematological dysfunction, APACHE II at the onset of bacteremia, and SAPS II at the onset of bacteremia were the main risk factors associated with death. Based on multiple logistic regression analysis, the independent predictor for death was renal dysfunction (OR 5.5; CI 1.3–22.4) [**Table 3**].

**Table 3:** Risk factors for in-hospital mortality.

	Non-survivorsN = 33	SurvivorsN = 20	p-value	Univariate analysisOR (95% CI)	Multivariate analysisOR (95% CI)
Mean age ± SD	69.55±18.22	56.35±18.58	0.016		1.03 (0.99–1.07)
Male gender	19 (57%)	9 (45%)	0.374	0.60 (0.2–1.84)	
Respiratory dysfunction	26 (78%)	13 (65%)	0.471	1.6 (0.45–5.66)	
Cardiovascular dysfunction	22 (66%)	8 (40%)	0.58	3 (0.95–9.48)	
Renal dysfunction	27 (82%)	9 (45%)	0.005	5.5 (1.58–19.17)	5.5 (1.3–22.4)
Hematologic dysfunction	23 (70%)	4 (20%)	<0.001	9.2 (2.45–34.56)	
Liver dysfunction	10 (30%)	2 (10%)	0.87	3.9 (0.76–20.15)	
Conservative method	12 (36%)	4 (20%)	0.208	2.28 (0.62–8.43)	
Adequate antimicrob ther 1st 24 h	12 (36%)	10 (50%)	0.329	0.57 (0.18–1.76)	
Septic shock	28 (85%)	10 (50%)	0.006	5.6 (1.54–20.42)	
Charlson >3	19 (57%)	7 (35%)	0.854	1.1 (0.36–3.40)	
APACHEII bacteremia, mean ± SD	16.70±4.09	13.35±4.51	0.01		1.1 (0.93–1.33)
SAPS II bacteremia, mean ± SD	44.30±11.51	37±13.83	0.04		1.0 (0.95–1.07)
SOFA bacteremia, mean ± SD	7.70±3.91	5.85±4.27	0.120		
Prior ICU admission	23 (69%)	10 (50%)	0.152	2.3 (0.73–7.25)	

CVC = central venous catheter; SAPS II = simplified acute physiology score; SOFA = Sepsis-related Organ Failure Assessment; APACHE II = Acute Physiology and Chronic Health disease Classification System II, Charlson = comorbidity index.

## Discussion

Our study showed no difference in mortality rates of patients with CR-BSI when the two methods of diagnosis are compared –standard vs. conservative (57% vs. 75%, p = 0.208), but there is a difference in mortality when the conventional method is compared to the conservative method in cases where the CVC is kept in place for more than 24 hours (56% vs. 100%, respectively).

The conservative method for the diagnosis of CR-BSI has been reported as reliable by Bouza et al [6] in a prospective study comparing techniques that keep the CVC in place during the diagnostic investigation of CR-BSI in ICU patients with short-term catheters. The search for conservative methods to diagnose CR-BSI in ICU patients is justified, since this is one of the major nosocomial infections. Prior studies have shown that approximately 70% of the CVCs removed for diagnostic purposes were not causal factors of infection [2].

In a metanalysis by Safdar et al eight different methods used to diagnose CR-BSI were assessed; the authors showed that the paired quantitative blood culture (conservative method) was the most accurate test, with a sensitivity of 0.87 [95% CI, 0.83–0.91] and a specificity of 0.98 [95% CI, 0.97–0.99] [8]. However, due to its cost and complexity, this method is not yet routinely used. On the other hand, the conservative method based on differential time to positivity was shown by Blot et al. to have 91% specificity and 94% sensitivity, with the advantage that many labs currently use automated continuous blood culture monitoring, which makes this method easy to perform and less expensive than the paired quantitative culture [17]. For all cases in our conservative group the diagnosis was performed by differential time to positivity.

The different mortality rates observed in our study and in the US SCOPE [18] (Surveillance and Control of Pathogens of Epidemiologic Importance) prospective analysis, 62% vs 27%, may be explained by the fact that the infections recorded in the US SCOPE study occurred both in ICU and non-ICU patients, whereas in our study all the infections occurred in ICU patients, who suffer from more complex and severe conditions. Another explanation is provided by the microbiologic analyses, since our gram-negative bacilli showed higher rates of resistance to ciprofloxacin, ceftazidime and imipenem and because in our series *Acinetobacter baumannii* was the most frequent gram-negative bacillus. A recent study [19] performed in our ICU to decrease mortality in septic shock and severe sepsis by applying a sepsis bundle, showed that the implementation of a rapid response team contributed to decreasing our mortality rates from 52% in 2005 to 16% in 2009, justifying our higher mortality rate compared with other studies [20] [21].

Another important difference was the rate of fungal infections, 36% vs. 7.6% in the US SCOPE project. Concerning gram-positive cocci, our infections were mostly caused by coagulase-negative staphylococci (54%) vs 58.9% in SCOPE.

Concerning *Staphylococcus aureus*, we had no infection caused by methicillin-resistant staphylococcus (MRSA) vs 30.7% of MRSA in SCOPE; and we had no vancomycin-resistant enterococcus vs 7.5% in SCOPE.

On other hand a recent multicenter Brazilian study showed that in Brazil our epidemiology is different from the typical epidemiology of ICU CR-BSI's. More than 50% of the all nosocomial bloodstream infections in this study were due to gram negative bacilli [22]. The Brazilian SCOPE study also discloses a pattern of BSI in Brazilian hospitals, considerably different from the American experience. A very high proportion of aerobic gram-negative bacteria, very high rates of resistance to carbapenems by non-fermentative gram-negative bacteria, higher mortality rates and a shift to non-albicans species of *Candida* were noted, and such findings may help Brazilian hospitals to develop their own guidelines for the treatment of BSI infections [22].

One of the major controversies, at present is about the best day for the measurement of severity scores (e.g. APACHE II); depending on the day, this may be a confounding factor when analyzing results in patients with infections. Thom et al [23] have shown in a retrospective study that time-adjusted hospital mortality correlates with the day chosen for the measurement of severity scores in patients with gram-negative bloodstream infections and the day of onset of bacteremia seems to be the best choice for these measurements. The same was observed in this study, there was a statistically significant difference between survivors and non-survivors from CRBSI concerning APACHE II and SAPSII scores measured on the day of onset of bacteremia (in univariate analysis).

Our study was not an interventional study but an observational study. We included only patients with CRBSI and our intention in this study was to alert ICU doctors regarding the decision to keep the catheter in place if there was a rapid time to positivity in blood cultures. For this purpose there is a need for very good communication between microlab and ICU doctor for a quick notification regarding blood culture results so they can remove the catheter as soon as they have the results.

To our knowledge, our study is the first assess the difference in outcome between ICU patients with CR-BSI diagnosed by standard versus conservative methods.

### Strengths and limitations

The main limitations of our study were that it was a retrospective study, conducted at a single center, on a small sample of patients. Although there was no difference in mortality rates, this small number of cases could lead to a type II error. However, despite these limitations, the study included a highly selective sample of patients with CR-BSI, and that 75% vs. 57% may be clinically relevant. Of the 16 cases of CR-BSI diagnosed using the conservative method, there was 100% of mortality when the CVC was kept in place for more than 24 hs. Thus we did not feel that the method of diagnosis is only a marker of sicker patients. Also we are not sure if a power analysis is important considering the context.

### Conclusions

In case of CR-BSI it can be expected that prompt catheter removal will result in shorter duration of BSI and improved outcomes [24], [25]. In our study there was a no statistically significant difference between the standard and conservative methods in-hospital mortality but there was a trend toward higher mortality rates among patients with CR-BSI diagnosed by the conservative method when the CVC was kept in place for more than 24 h. Further studies should be conducted to confirm this hypothesis.
